# Caspase cleavage of iASPP potentiates its ability to inhibit p53 and NF-κB

**DOI:** 10.18632/oncotarget.6478

**Published:** 2015-12-05

**Authors:** Ying Hu, Wenjie Ge, Xingwen Wang, Gopinath Sutendra, Kunming Zhao, Zinaida Dedeić, Elizabeth A. Slee, Caroline Baer, Xin Lu

**Affiliations:** ^1^ The School of Life Science and Technology, Harbin Institute of Technology, Harbin, China; ^2^ Ludwig Institute for Cancer Research Ltd., Nuffield Department of Clinical Medicine, University of Oxford, Oxford, UK

**Keywords:** iASPP, caspase, p53, RelA/p65

## Abstract

An intriguing biological question relating to cell signaling is how the inflammatory mediator NF-kB and the tumour suppressor protein p53 can be induced by similar triggers, like DNA damage or infection, yet have seemingly opposing or sometimes cooperative biological functions. For example, the NF-κB subunit RelA/p65 has been shown to inhibit apoptosis, whereas p53 induces apoptosis. One potential explanation may be their co-regulation by common cellular factors: inhibitor of Apoptosis Stimulating p53 Protein (iASPP) is one such common regulator of both RelA/p65 and p53. Here we show that iASPP is a novel substrate of caspases in response to apoptotic stimuli. Caspase cleaves the N-terminal region of iASPP at SSLD294 resulting in a prominent 80kDa fragment of iASPP. This caspase cleavage site is conserved in various species from zebrafish to *Homo sapiens*. The 80kDa fragment of iASPP translocates from the cytoplasm to the nucleus via the RaDAR nuclear import pathway, independent of p53. The 80kDa iASPP fragment can bind and inhibit p53 or RelA/p65 more efficiently than full-length iASPP. Overall, these data reveal a potential novel regulation of p53 and RelA/p65 activities in response to apoptotic stimuli.

## INTRODUCTION

Many signaling proteins seem to have opposing and/or cooperative biological functions. One example is the DNA damage- or infection-induced expression of the tumour suppressor p53 and the inflammatory mediator RelA/p65 (a subunit of NF-κB). Alterations in gene expression mediated by these transcription factors often results in opposing cellular outcomes: activation of p53 often leads to cell cycle arrest or apoptosis [1], whereas RelA/p65 activation generally induces pro-proliferative and anti-apoptotic signaling. Constitutively activated RelA/p65 is associated with initiation and development of multiple cancers and is also associated with drug resistance [2]. Several studies have reported cooperative effects between p53 and RelA/p65 signaling pathways in regulating apoptosis. For example, RelA/p65 activation mediates p53-dependent apoptosis in SAOS2 cells [3], and p53-dependent RelA/p65 activation is required for doxorubicin and etoposide-induced cell death in neuroblastoma cells [4]. A major question is how p53 and RelA/p65 signaling is intertwined, particularly during the apoptotic response.

The evolutionarily conserved Ankyrin repeat domain, SH3 domain and Proline rich sequence containing Protein (ASPP) [5-8] family is known to interact with p53 and RelA/p65 and thus represents a group of regulators that might mediate links between p53 and RelA/p65 signalling. The most well-known function of the ASPP family of proteins is the ability of ASPP1 and ASPP2 to stimulate p53-mediated apoptosis and the ability of iASPP to inhibit this process [5, 6]. The oncogenic potential of iASPP has been highlighted by increased mRNA and protein levels in various human tumours, resulting in drug resistance in breast, hepatocellular, ovarian malignancies and leukemia [6, 9-13].

In addition to regulating p53, iASPP has also been shown to bind RelA/p65 and inhibit its transcriptional activity *in vitro* [7]. Consistent with this, iASPP regulation of human immunodeficiency virus type 1 (HIV1) expression is largely dependent on RelA/p65 [14]. In C57BL6 mice, iASPP deficiency resulted in enhanced RelA/p65 transcriptional activity with increased expression of its target *ICAM1* in mouse vascular endothelium [15]. However, in the epidermis of 129/C57BL6 background mice, iASPP deficiency failed to cause a detectable increase in RelA/p65 activity [16]. Thus, the biological importance of the iASPP-RelA/p65 interaction remains unclear.

Although iASPP has an anti-apoptotic function by inhibiting p53 family members [6, 17], iASPP has also been shown to promote apoptosis under certain circumstances. For example, iASPP inhibition of RelA/p65 stimulates DNA damage-induced apoptosis in non-malignant lymphocytes and fibroblasts [18]. The pro-apoptotic function of iASPP has also been associated with the stabilization of p73 [19]. These studies suggest that under certain conditions iASPP may have pro- or anti-apoptotic effects, depending on its regulation of p53 or RelA/p65, respectively.

Endogenous full-length iASPP is mainly present as a homo-oligomer in the cytoplasm and the N-terminus of iASPP has been shown to be required for its cytoplasmic localization [20]. Phosphorylation of serine resides 84 and 113 at the N-terminus prevents a N- and C-terminal self-interaction and reveals both the p53 interaction site at the C-terminus and the nuclear localization RaDAR code (RanGDP and Ankyrin Repeats binding code), which enables its nuclear entry via the recently identified RaDAR nuclear import pathway [21]. The RelA/p65 interaction site is also at the C-terminus of iASPP [7], therefore RelA/p65 inhibition may be more responsive to nuclear than cytoplasmic iASPP, similar to the situation for iASPP-mediated p53 inhibition. Although phosphorylation of the N-terminus of iASPP is the only identified mechanism for its nuclear translocation, it is possible that cleavage of the N-terminus could also represent a phosphorylation-independent mechanism for iASPP nuclear translocation.

In response to apoptotic stimuli, caspases (cysteine aspartic acid-specific proteases) are the most prominent proteolytic enzymes responsible for protein cleavage [22, 23]. Although caspase cleavage was initially considered to be the end result of a physiologic or pathologic apoptotic stimuli, further findings have suggested that caspases can cleave specific proteins [32] and the cleaved fragments can positively or negatively regulate apoptosis [24-30]. In this study, we examined whether iASPP is a substrate for caspases and if proteolytic cleavage of iASPP can regulate the transcriptional activity of p53 and RelA/p65.

## RESULTS

### Induction of apoptosis induces caspase-mediated cleavage of iASPP

**Figure 1:**
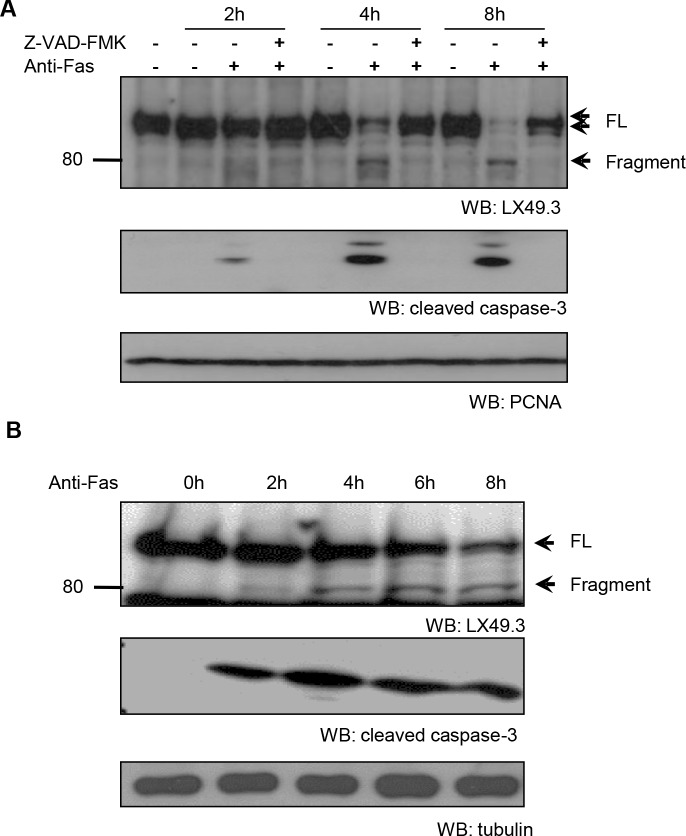
Caspase-dependent iASPP cleavage in anti-Fas antibody treated CEM and Jurkat cells CEM **A.** or Jurkat **B.** cells were exposed to anti-Fas antibody (250ng/mL) for the indicated period of time. To inhibit caspase activity, cells were pre-incubated with 25μM z-VAD-FMK for 1 hour. iASPP cleavage was detected by western blotting with LX49.3 antibody. Caspase cleavage was validated by western blotting with anti-cleaved caspase-3 antibody. PCNA or β-tubulin was used as the loading control. FL, full length; WB, western blot.

To investigate if iASPP could be a potential substrate of caspase during the apoptotic response, we used an anti-Fas antibody to trigger apoptosis in human lymphoid tumour CEM cells that contain constitutively activated RelA/p65 and mutant p53 [31]. The iASPP protein was subsequently analyzed by immunoblotting with the LX49.3 antibody, which is directed towards the middle region of iASPP (Supplementary Figure S1A). Within 2 hours of anti-Fas treatment, a fragment of iASPP (∼80kDa) was observed and the accumulation of this fragment was prevented by treatment with the general caspase inhibitor z-VAD-FMK (Figure 1A). This suggests that the 80kDa iASPP fragment is a result of caspase-mediated cleavage from the ∼100kDa full-length iASPP. This result was confirmed with an antibody against the C-terminus of iASPP (LX142.3; Supplementary Figure S1B).

Proteolytic cleavage of iASPP could also be detected when apoptosis was induced by an anti-Fas antibody in the Jurkat leukemic T-cell line (Figure 1B) and in staurosporine- or etoposide-treated Jurkat cells (Supplementary Figure S1C and S1D). Quantification showed that changes in the levels of the 80kDa fragment of iASPP were associated with similar changes in cleaved caspase-3 levels (Supplementary Figure S1E-H), suggesting that caspase-3 might be one of the caspases responsible for the generation of the 80kDa iASPP fragment.

### iASPP can be cleaved by caspases at D91 and/or D294 in its N-terminus

Using a cell-free system in which caspase is activated in the cytosol by addition of dATP and cytochrome c [32], we examined cleavage of endogenous iASPP (protein in the cell free cytosol) or exogenous iASPP (*in vitro* translated iASPP added into cell free system) (Supplementary Figure S2). Activated cleaved caspase-3 and the 80kDa iASPP fragment were observed after stimulation of caspase, in a time-dependent manner (Supplementary Figure S2A). The 80kDa iASPP fragment was only detected when iASPP was tagged at the C-terminus, not the N-terminus, suggesting that the 80kDa fragment derives from the C-terminus.

**Figure 2:**
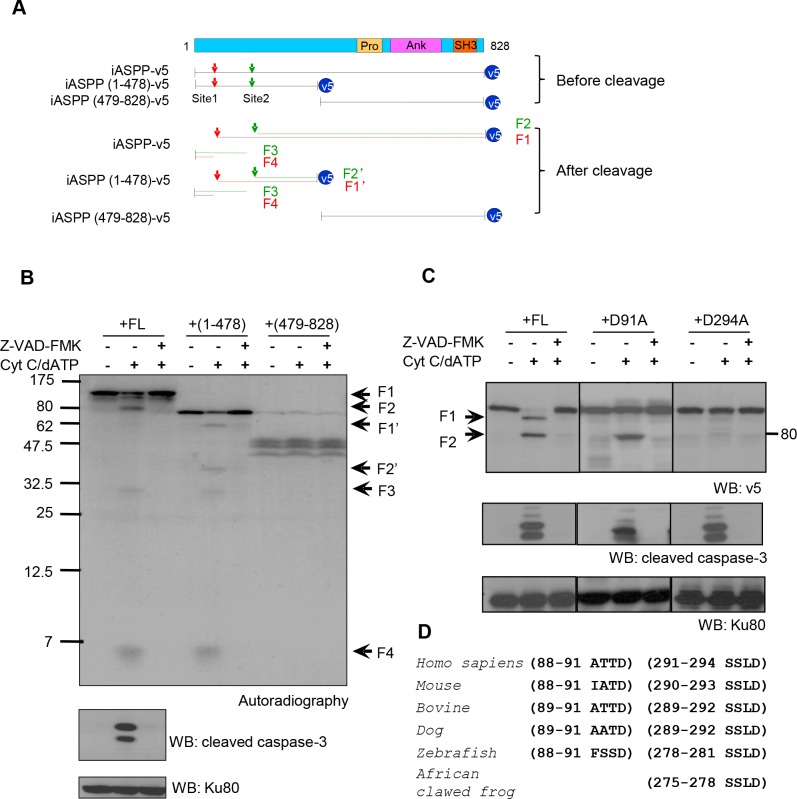
Putative iASPP cleavage sites at D91 and D294 **A.** Schematic representation of iASPP, truncated iASPP and the predicted iASPP caspase cleavage products. At the top of the diagram, FL-iASPP is schematically represented with its functional domains. Below this, the FL-iASPP-v5 and iASPP truncated products are shown as lines. The arrowheads indicate the potential iASPP cleavage sites responsible for the production of 95kDa (red) and 80kDa (green) iASPP fragments from FL-iASPP, or 65kDa (red) and 50kDa (green) from (1-478)-iASPP. SH, Src homology domains; Pro, proline rich domain; Ank, ankyrin repeats. **B.** FL-iASPP, iASPP(1-478), and (479-828) were *in vitro* translated and labelled with [35S] methionine and mixed with cell-free extracts in the presence or absence of Cytochrome c (Cyt C) and dATP. The presence of radiolabelled iASPP and iASPP fragments were detected by autoradiography. Ku80 was used as a loading control. **C.** Two possible *in vitro* caspase cleavage sites contributing to the generation of the 95kDa and 80kDa iASPP fragments were mutated from aspartate (**D**) to alanine (**A**). FL-iASPP and the iASPP mutants were translated *in vitro*. The same amounts of product were added into apoptosis triggered cell-free extracts. z-VAD-FMK was added as indicated to inhibit caspase activity. The proteins were probed with anti-v5 antibody. The arrowheads indicate iASPP and cleaved iASPP, as in panel **A.** Cleaved-caspase-3 was used as a marker for caspase activation. Ku80 was used as a loading control. **D.** Alignments of the consensus caspase cleavage site in iASPP among different species. WB, western blot.

To identify the caspase cleavage site(s), *in vitro* translated 35S-labeled full length (FL), N-terminus (1-478) or C-terminus iASPP (479-828) were assessed in the cell-free system. Cleaved fragments were detected for FL-iASPP and iASPP (1-478), but not for iASPP (479-828), suggesting that the cleavage sites are located at the N-terminus (Figure 2A-2B and Supplementary Figure S3). Four major cleavage products of approximately 95, 80, 30, and 5 kDa (numbered F1-4 in Figure 2B, lanes 1-3) were generated from FL-iASPP by activated caspases. Similarly, four major cleaved fragments were generated from iASPP-(1-478) (numbered F1′-2′, F3-4, Figure 2B and Supplementary Figure S3, lanes 4-6). These data suggest that there are two caspase cleavage sites in iASPP and that the N-terminus of iASPP is required for caspase cleavage.

On the basis of the size of the *in vitro* generated fragments and the known consensus sequences for caspase cleavage, we identified two potential cleavage sites in iASPP, positioned after aspartic acids (D) 91 at the motif ATTDD and 294 at the motif SSLD. We generated two mutants, carrying an alanine (A) substitution at D91 or D294, and exposed these to apoptotic cell-free extracts. The D294A mutation prevented generation of the 80kDa fragment, and both mutations prevented generation of the 95kDa fragment (Figure 2C). Therefore, although the 95kDa fragment results from cleavage at D91, D294 is also required for this specific cleavage. The cleavage reaction is summarized in Figure 2A.

The 80kDa iASPP fragment was detected in the cell-free extracts and in cell cultures after anti-Fas antibody treatment, whereas the 95kDa band could only be detected *in vitro*. Therefore cleavage at D91 is either unlikely to occur *in vivo*, or the 95kDa fragment is further cleaved into a 80kDa fragment or rapidly degraded. Interestingly, the cleavage sequence SSLD294 is conserved in many species (Figure 2D), whereas ATTD91 is not, suggesting the cleavage at SSLD294 may be more biologically relevant.

### Caspse-3 activation contributes to iASPP cleavage

**Figure 3:**
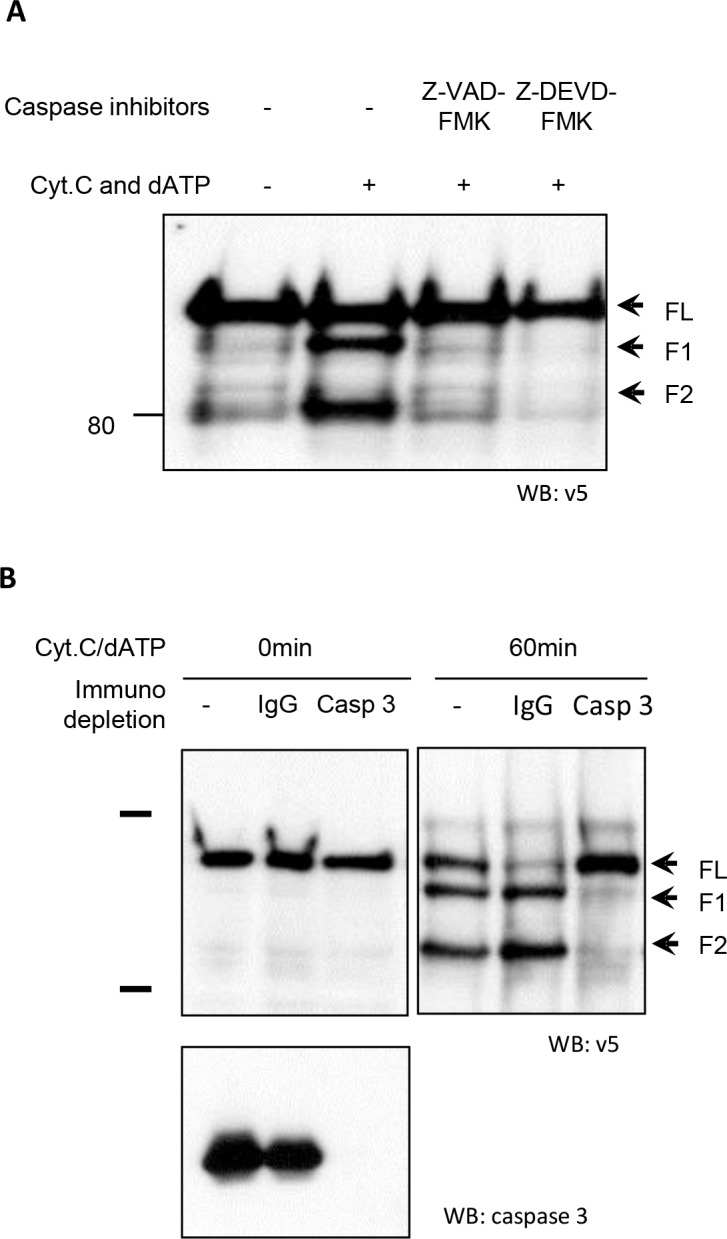
Abrogation of iASPP cleavage in CEM cell-free extracts by caspase-3 depletion **A.** CEM cell free extracts were treated with a pan-caspase inhibitor (Z-VAD-FMK) or specific caspase-3 inhibitor (Z-DEVD-FMK) 1 hour before triggering caspase cascade by adding cytochrome c and dATP. The cleavage of iASPP was detected by anti-v5 antibody. **B.** Caspase cascades were triggered at 37°C by adding Cyt C and dATP in the control, IgG depleted or caspase-3 depleted CEM cell-free extracts containing same amount of *in vitro* translated v5 tagged iASPP. Mixtures were separated by 8% SDS-PAGE followed by immunoblotting with the anti-v5 antibody. Immunodepletion efficiency was confirmed by western blotting with anti-caspase-3 antibody. The arrowheads indicate iASPP and cleaved iASPP fragments.

iASPP cleavage in the *in vitro* cell-free system was blocked by pre-treatment with a specific caspase-3 inhibitor (Z-DEVD-FMK), suggesting that iASPP is a potential caspase-3 substrate (Figure 3A). When CEM cell free extracts were depleted of caspase-3 using an anti-caspase-3 antibody (Figure 3B), iASPP cleavage was completely abrogated, whereas cleavage was observed in untreated and IgG-treated (control) CEM cell free extracts after incubation with cytochrome c and dATP (Figure 3B). This suggests that iASPP cleavage is dependent on caspase-3 activation *in vitro*.

### Cleaved iASPP fragments predominantly locate in the nucleus

**Figure 4:**
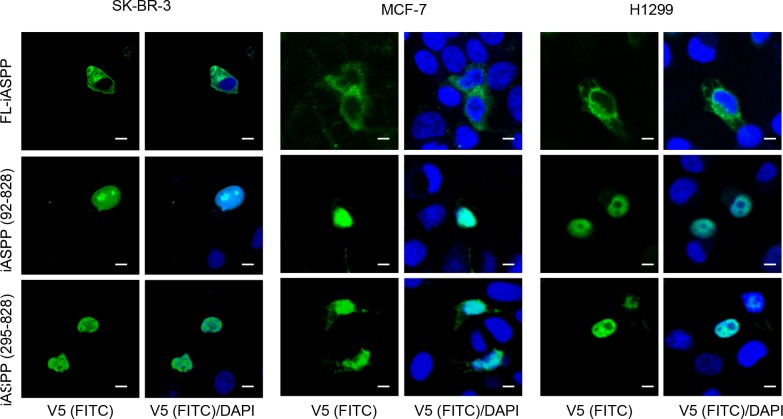
Cleaved iASPP fragments were localized at nucleus Shown are confocal microscopy images of SK-Br-3, MCF-7 and H1299 cells, fixed with 4% PFA 24-48 hours after transfection with FL-iASPP, iASPP (92-828) and (295-828)-v5. Protein localization was detected by mouse monoclonal anti-v5 antibody, followed by the anti-mouse FITC-conjugated secondary antibody. DAPI shows the location of nuclei. Scale bars 10 μm.

As nuclear localization is important for iASPP mediated inhibition of p53 and most-likely of RelA/p65 as well, we investigated if the caspase-generated C-terminus iASPP fragments are localized in the nucleus. Truncated V5-tagged iASPP mutants were generated and transfected into SK-Br-3 cells (which have p53 mutation and NF-κB constitutive activation), H1299 cells (p53-null, low NF-κB activity) and MCF-7 cells (p53-wild type with elevated NF-κB activity) (Figure 4). In all three cell types FL-iASPP mainly localized in the cytoplasm, whereas truncated iASPP - both iASPP(92-828) and iASPP(295-828) - localized exclusively in the nucleus (Figure 4). Nuclear translocation of truncated iASPP was independent of p53 and RelA/p65 status (Figure 4). These data suggest that iASPP is cleaved by cytosolic caspases and the stable 80kDa iASPP fragment accumulates in the nucleus.

### iASPP(295-828) fragment binds better to p53 and RelA/p65 than full length iASPP

**Figure 5:**
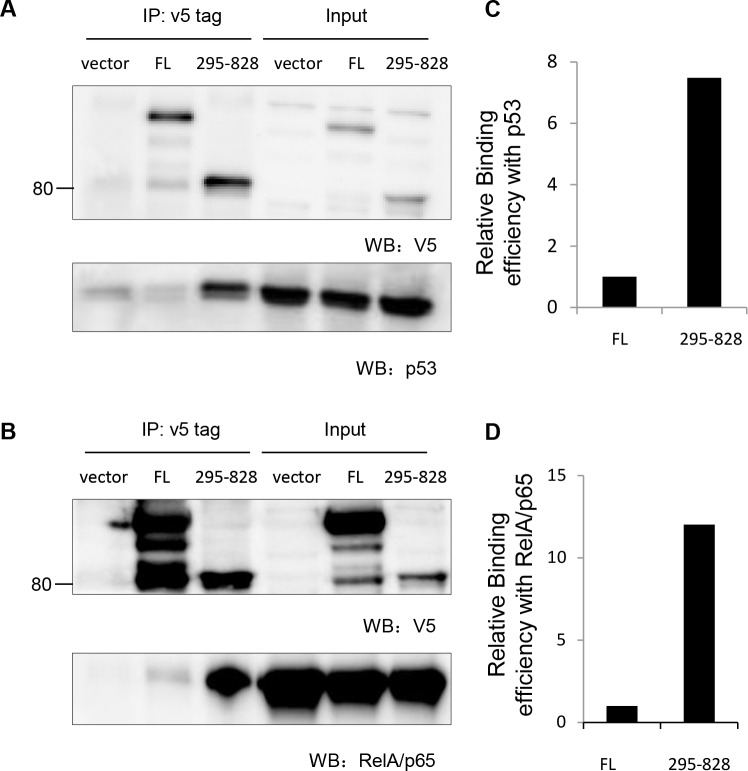
Cleaved iASPP fragments formed stable complex with p53 and RelA/p65 **A.** 293T cells were transfected with p53 together with either empty vector control plasmid, FL- iASPP or iASPP (295-828). Exogenous iASPP was immunoprecipitated by anti-v5 antibody (upper panel) and co-precipitated p53 was probed by anti-p53 antibody (lower panel). **B.** 293T cells were transfected with RelA/p65 NF-κB together with either empty vector control plasmid, FL- iASPP or iASPP (295-828). Exogenous iASPP was immunoprecipitated by anti-v5 antibody (upper panel) and co-precipitated RelA/p65 was probed by anti-p65 antibody (lower panel). **C**.-**D**. Quantification data of the relative amount of p53 or RelA/p65 that co-precipitated with per arbitrary unit of FL-iASPP or iASPP (295-828), based on the western blots in panels **A**. and **B**.

To address the potential biological relevance of the nuclear-localized 80kDa iASPP fragment, we first assessed the binding of FL-iASPP and the 80kDa iASPP fragment to p53 and RelA/p65. We detected p53 and RelA/p65 in the iASPP(295-828) anti-V5 immunoprecipitate, suggesting that p53 and RelA/p65 can form a stable complex with iASPP(295-828) in cells (Figure 5A-5B). Furthermore, ∼7.5-fold and 12-fold less p53 and RelA/p65, respectively, was co-precipitated with FL-iASPP (despite similar or more FL-iASPP immunoprecipitate with the anti-v5 tag antibody) (Figure 5C and 5D). These data suggest that the 80kDa iASPP fragment may be more efficient at binding p53 and RelA/p65 than FL-iASPP.

### Cleaved iASPP fragments inhibit p53 and RelA/p65 transcriptional activity more efficiently than the full length iASPP

**Figure 6:**
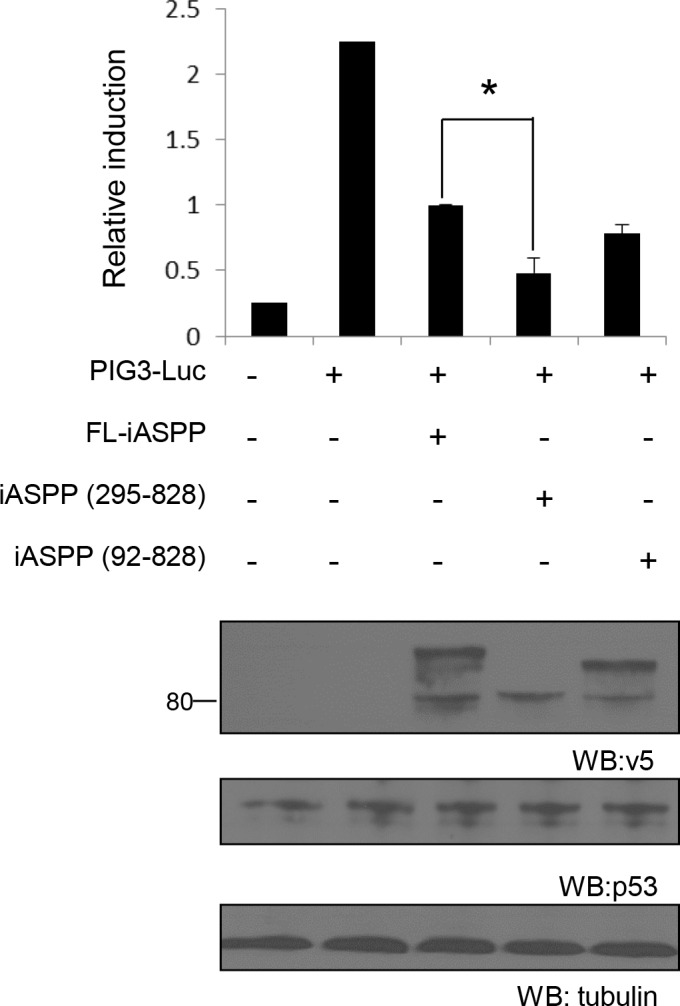
Cleaved iASPP fragments more efficiently inhibit the transcriptional activities of p53 than FL-iASPP A total of 200 ng of p53-dependent luciferase PIG3 was cotransfected into MCF-7 cells. FL- iASPP, iASPP (295-828) or (92-828) were transfected together with luciferase reporter as indicated. Total DNA for each transfection was made up to 500 ng by using vector plasmid. Luciferase activities were measured at 36-48 h after transfection and are presented as the increase in activation over reporter alone. Data shown are the mean of three independent experiments, and standard deviations are shown (error bars). *P < 0.05. The protein levels of iASPP and iASPP fragments were detected by western blotting with the anti-tag (v5) antibody (lower panels).

To assess whether fragmented iASPP can inhibit p53 and RelA/p65 transcriptional activity, we first used a p53-inducible gene 3 (PIG3) reporter assay in the breast cancer cell line MCF-7, which contains wild-type p53. FL-iASPP reduced p53 transcriptional activity by ∼55% at the PIG3 reporter (*P* < 0.01, Figure 6). iASPP(295-828) and iASPP(92-828) inhibited the transcriptional activity of p53 more efficiently than FL-iASPP (∼79% and ∼65%, respectively, *P* < 0.05) reduction in activity, respectively; Figure 6). The inhibitory effect of iASPP(295-828) on p53 transcriptional activity was ∼1.5 fold higher than that of FL-iASPP (*P* < 0.05, Figure 6).

**Figure 7:**
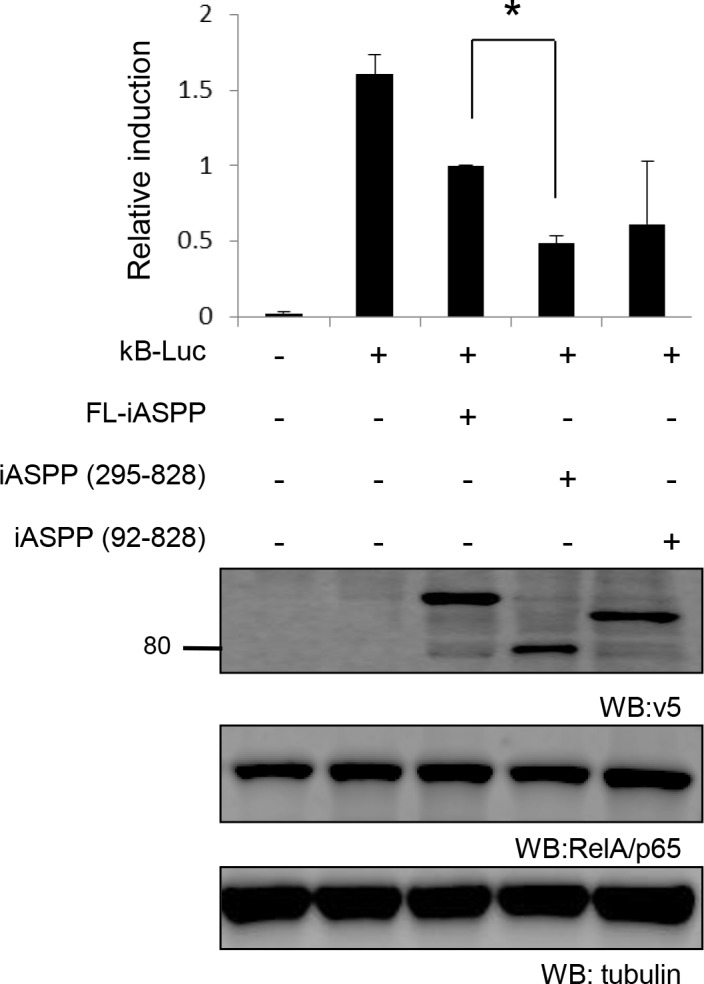
Cleaved iASPP fragments more efficiently inhibit the transcriptional activities of RelA/p65 than FL-iASPP A total of 200 ng of reporter κB -dependent luciferase was cotransfected into SK-Br-3 cells. FL-iASPP, iASPP (295-828), or (92-828) were transfected together with luciferase reporter as indicated. Total DNA for each transfection was made up to 500 ng by using vector plasmid. Luciferase activities were measured at 36-48 h after transfection and are presented as the increase in activation over reporter alone. Data shown are mean of three independent experiments, and standard deviations are shown (error bars). *P < 0.05. The protein levels of iASPP and iASPP fragments were detected by western blotting with the anti-tag (v5) antibody.

Constitutive activation of RelA/p65 has been reported in breast cancer [33], so we compared the inhibitory effect of FL-iASPP, iASPP(295-828) and iASPP(92-828) on RelA/p65 transcriptional activity in the breast cancer cell line SK-Br-3, in which RelA/p65 is constitutively activated [34, 35]. FL-iASPP reduced RelA/p65 transcriptional activity by ∼38% (*P* < 0.01) compared to the control (Figure 7). Under the same conditions, iASPP(295-828) and iASPP(92-828) reduced NF-κB reporter activity more substantially: ∼70% (*P* < 0.05) and ∼62% (*P* < 0.05), respectively (Figure 7). The inhibitory effect of iASPP(295-828) on RelA/p65 transcriptional activity was ∼2-fold higher than FL-iASPP (*P* < 0.05) (Figure 7). These results indicate that cleavage at D294 produces an iASPP fragment that can efficiently inhibit RelA/p65 or p53 activity.

## DISCUSSION

**Figure 8:**
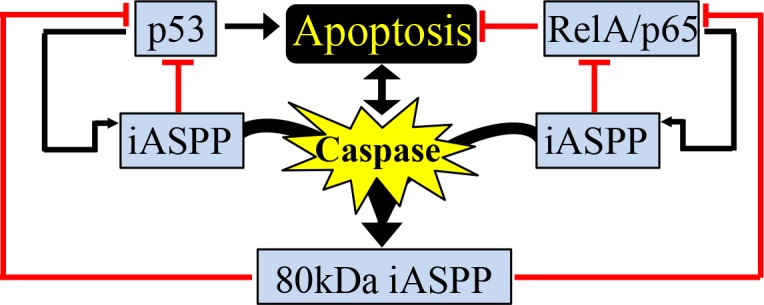
Proposed model caspase-mediated iASPP cleavage in regulation of p53 and/or RelA/p65 activities Schematic outlining caspase-mediated generation of the 80kDa iASPP fragment, which results in the inhibition of p53 and RelA/p65 transcriptional activity. Many feedback loops are present as p53 and RelA/p65 can regulate the expression of iASPP. Both full-length iASPP and the 80kDa iASPP fragment can inhibit p53 and RelA/p65 transcriptional activity. See Discussion for more detailed explanations.

Here we show iASPP is a novel caspase substrate. In response to apoptotic stimuli, iASPP is cleaved in a p53- and RelA/p65-independent manner by caspases at D294 to generate an 80kDa iASPP fragment. The cleaved 80kDa iASPP fragment mainly localizes to the nucleus and is a more efficient binding partner and a more potent inhibitor of p53 and RelA/p65 than the full-length iASPP. This work identifies a novel pathway by which caspases may be able to negatively or positively regulate apoptosis *via* iASPP-mediated p53 or RelA/p65 inhibition, respectively (Figure 8) [36].

Caspases and iASPP are highly conserved proteins found from *C. elegans* to *Homo sapiens* [6, 37], suggesting they may be important components of the cell's apoptotic machinery. Importantly, caspase cleavage has been widely accepted as apoptosis regulatory mechanisms. The iASPP SSLD-294 motif is conserved in various species from zebrafish to *Homo sapiens*, suggesting that the identified caspase-mediated regulation of iASPP activity may be conserved in other organisms.

It is important to note that caspase-3 can initiate a cascade of apoptotic responses by initiating the cleavage of several proteins implicated in apoptosis. For example, caspase-3-mediated cleavage of retinoblastoma, X-linked inhibitor of apoptosis protein (XIAP) and mediator of DNA damage checkpoint protein are all important for initiating the apoptotic response [27, 28, 38]. Alternatively, caspase-3 activation can also signal to anti-apoptotic pathways. For example, Bim degradation by caspase-3 attenuates apoptosis in osteoclasts [29, 30]. In a similar manner, the caspase-3 cleaved 80kDa iASPP fragment may also have pro- or anti-apoptotic effects depending on its regulation of p53 or RelA/p65, respectively. Although we limited our investigation to caspase-3, it is feasible that other caspases may also regulate iASPP cleavage. For example, the SSLD motif has previously been reported to be a caspase-8 motif [39]. Caspase-8 is one of the initiator caspases that is activated upon Fas binding to its ligand (anti-Fas) in the death-receptor pathway, suggesting that this caspase may also be a candidate for the cleavage of iASPP.

The cleaved iASPP fragments, iASPP(92-828) and iASPP(295-828), lack the N-terminus required for iASPP dimerization and are predominantly nuclear. This is in agreement with our previous findings that the N-terminus of iASPP is required for its cytoplasmic localization [20] and that phosphorylation of the N-terminal Ser84 by cyclin B1/CDK1 on iASPP disrupts iASPP dimerization [11] resulting in the exposure of the recently identified RaDAR code located at its C-terminal Ankyrin repeats, which enables its nuclear entry via the RaDAR nuclear import pathway[21]. Recently, the precise intermolecular interaction of iASPP has also been dissected by Iosub-Amir et al [40], where they show that the N-terminal iASPP peptides 60-74 and 540-562 have the strongest intermolecular interactions with the C-terminal ankyrin-repeat and SH3 domain. Our work is in keeping with this observation as caspase cleavage would remove the 60-74 residues of N-terminal iASPP important for dimerization and cytoplasmic retention, thereby facilitating its nuclear entry. Therefore, we provide an alternative mechanism for the nuclear localization of iASPP and subsequent inhibition of the p53 and RelA/p65 transcriptional activity.

It has been reported that activated p53 and RelA/p65 can induce iASPP expression [10, 36, 41], suggesting iASPP may be a direct transcriptional target of p53 and RelA/p65. Therefore, it is interesting to speculate that iASPP might regulate p53 and RelA/p65 through negative feedback loops (Figure 8). The biological outcomes resulting from p53 and RelA/p65 activation are complex and are stimulus- and cell context-dependent. RelA/p65 can selectively interact with the evolutionarily conserved p53 polymorphism p53Pro72, but not with the late-evolved polymorphism p53Arg72, to enhance apoptosis. In response to DNA damage, p53Pro72 and RelA/p65 cooperatively induce the expression of Caspase 4/11 and other inflammatory-related genes to induce apoptosis [42]. The molecular basis of this p53 polymorphic selection by RelA/p65 remains largely unknown. Interestingly, iASPP was also reported previously to selectively bind p53Pro72 to inhibit p53-mediated apoptosis [43]. It is therefore tempting to speculate that the identified apoptotic response mediated by iASPP-p53 and iASPP-RelA/p65 may be more prominent in cells expressing p53Pro72. Since the p53 polymorphism at codon 72 is a common polymorphism with a distinct distribution depending on ethnicity and latitude, the identified iASPP-p53 and iASPP-RelA/p65 interactions might produce negative or positive feedback signals to regulate apoptosis progression.

In addition, structural and biological data from Iosub-Amir et al. provide an alternative explanation for the opposite biological outcomes produced by the ASPP family of proteins [40]. The intermolecular interaction of iASPP (anti-apoptotic) and ASPP2 (pro-apoptotic) are both mediated by their conserved proline-rich and ankyrin-SH3 domain. However, the precise binding sites are different, which suggests that the correct intermolecular interaction is critical for the apoptosis related biological activity of the ASPP proteins [40]. Furthermore, the cleavage of iASPP in the N-terminal proline-rich domain disrupts this intermolecular interaction with the ankyrin-SH3 domain and thus may affect its original anti-apoptotic biological activity.

Although we provide strong evidence between the binding of cleaved iASPP and inhibition of p53 and RelA/p65 transcriptional activity, future work needs to address the biological significance of this novel interaction and address under what conditions these feedback loops may occur and how they are able to initiate cellular outcomes such as apoptosis in response to physiologic and pathologic stimuli.

## MATERIALS AND METHODS

### DNA constructs

All of the iASPP constructs used in this study were obtained from human iASPP. pCDNA3.1-iASPP, pCDNA3.1- iASPP (1-478) and pCDNA3.1- iASPP (479-828) were previously described. pCDNA-D91A-iASPP and pCDNA-D294A-iASPP were generated by QuikChange® Site-Directed Mutagenesis Kit purchased from Stratagene according to instructions. pCDNA3.1- iASPP (92-828) and pCDNA3.1- iASPP(295-828) obtained by PCR. The resulting iASPP(92-828) and (295-828) PCR fragments were subsequently subcloned into the pCDNA3.1 vector by TA cloning.

### Cell cultures and apoptosis induction

Human T lymphocyte Jurkat cell was grown in RPMI1640 (GIBCO, Invitrogen cell culture, UK) supplemented with 10% fetal calf serum (FBS, PAA Laboratories), 2mM L-Glutamine and 100μg/ml penicillin G, 100mg/ml Streptomycin (GIBCO). CEM cell line was grown in RPMI 1640 complete medium containing 1% sodium pyruvate. Human breast cancer cells SK-Br-3 and MCF-7, human lung cancer cells, H1299 and human embryonic kidney cells 293T were grown in DMEM (GIBCO, Invitrogen cell culture, UK) supplemented with 10% fetal calf serum (FBS, PAA Laboratories), 2mM L-Glutamine and 100μg/ml penicillin G, 100mg/mL Streptomycin (GIBCO). All cultures were maintained in a humidified chamber in 5% CO_2_ atmosphere at 37°C. Cells in the exponential phase of growth were used for analysis. For induction of apoptosis, cells (1-3×10^5^/mL) were incubated with anti-Fas IgM monoclonal antibody (Upstate Biotechnology Inc.) at a concentration of 250ng/mL, for the time period indicated in the legend to each figure. Etopside and Staurosporine were also used to induce apoptosis in Jurkat cells at indicated concentrations for 4hours. To inhibit caspase activity, cells were pre-incubated with 25μM z-VAD-FMK for 1hour.

### Immunoblots

Cells were washed twice with ice-cold 1 × PBS, and then lysed in appropriate volumes of RIPA supplemented with protease and phosphatase inhibitors. Equal amounts of proteins were separated by SDS-PAGE and transferred to the nitrocellulose membrane. Proteins were detected by adding specific antibodies followed by secondary antibodies conjugated to HRP. The HRP signal was developed by using ECL. Blots were exposed to X-ray film for the appropriate time period to detect specific protein signals.

### *In vitro* translation of plasmids

*In vitro* transcription and translation of plasmids was performed using the Promega TNT T7 Quick coupled transcription/translation system following the manufacturer's instructions. The reaction components included 40μL TNT® Quick Master Mix, 1μL unlabeled methionine (for cold IVT) or 2μL [35S]-labeled methionine (for hot IVT), and 1μg plasmid. The reaction mixture was made up to a final volume of 50μL by adding appropriate nuclease free water. The reaction was incubated at 30°C for 1 hour.

### Preparation of “Wang-Type” cell-free extracts

Cells were harvested by centrifuging at 400g for 5 minutes at 4°C. The resulting cell pellet was washed twice in 50mL of ice-cold 1 × PBS and then transferred to a 2mL Dounce-type homogenizer and pelleted again, followed by adding two volumes of ice-cold WCEB. Cells were allowed to swell under the hypotonic conditions for 15 minutes on ice, and lysed with 20-100 gentle strokes using a B-type pestle. The cell lysate was transferred to an Eppendorf tube and centrifuged for 15 minutes at 15,000×g (4°C). To activate apoptosis in cell-free extact, reactions were performed in a 10μL volume including 5μL cell-free extracts (10-20mg/mL protein), 1μL of 500ng/μL cytochrome c (Promega), 1μL of 10mM deoxyadenosine-5-triphosphate (dATP) (Promega), and 3μL of WCEB. Neither cytochrome c nor dATP was added to the negative control, and were replaced by WCEB. All reactions were incubated at 37°C for 10-90 minutes. 5 × SDS-PAGE loading buffer was then added to stop the reactions. After boiling for 5 minutes at 95°C, the lysates were subjected to the indicated analysis.

### Immunodepletion assay

Lysates were immunodepleted of caspase-3 prior to induce caspase cascades by adding dATP and cytochrome c. Briefly, protein G sepharose beads (40μl of 50% slurry) were blocked with 10% FBS in DMEM for 1 hour, then pre-incubated on a rotary mixer at 4°C for 3 hours with 5μg of the indicated anti-caspase antibody, and thoroughly washed to remove unbound antibody. 100μL “Wang-Type” cell-free extract was then incubated with antibody coated beads overnight at 4°C. The immunodepleted supernatants were removed by centrifugation and subjected to further analysis. For all experiments, control reactions were performed with pre-immune rabbit serum instead of the anti-caspase serum.

### Immunoprecipitation

293T cells were transfected vector control, FL-iASPP or iASPP(295-828) together with same amount of p53 or RelA/p65 by using Fugene 6 as indicated in the figures. 48 hours after transfection, cells were harvested and subjected to an immunoprecipitation assay. Briefly, equal amount of protein lystates were incubated with specific anti-v5 tag antibody and protein A/G beads overnight at 4°C. The beads were washed three times in cold NETN buffer. The precipitated iASPP protein and co-precipitated p53 or RelA/p65 were examined by western blotting and visualized by anti-v5 and anti- p53 or RelA/p65 antibody, respectively.

### Immunofluorescence assay

Cells grown on cover slips in a 24 well plate were fixed in 4% paraformaldehyde solution for 20 minutes after three washes in pre-warmed 1 × PBS. After another round of washes in 1 × PBS, cells were permeabilised with 1% Triton X-100 solution on ice for 4 minutes. The permeabilisation solution was then removed by adding 1 × PBS, following which cells were blocked in 1% fish gelatin (G7765, Sigma-Aldrich) for 20 minutes. Incubation with the appropriate diluted primary antibody was performed for 1 hour at RT, followed by three washes in 1 × PBS for 15 minutes each. Cells were then incubated with the fluorescently labeled secondary antibody for 1 hour. Typically, these secondary antibodies were used at 1:400 dilutions in blocking solution. The secondary's were replaced with 1 × PBS and rinsed in H_2_O before being mounted by Mowiol onto slides. Images were captured by confocal microscopy.

### Transactivation assay

Transactivation assays were modified from the protocol described before [5]. Briefly, the same amount PIG3- or κB-luciferase reporter together with either full length iASPP (1-828), iASPP (295-828), or (92-828) plasmids were transfected into MCF-7 or SK-Br-3 cells, respectively. Each of transfection was included the same amount of Renilla, which was used to standardize transfection efficiency. Vector plasmid was added to samples, when necessary, to bring the DNA to an equal amount. At 36-48 h after transfection, the luciferase activities in cell lysates were measured with the luciferase assay system (Promega) and presented as the increase in activation over reporter alone.

## SUPPLEMENTARY MATERIAL FIGURES


