# Supplementary material for: Caspase cleavage of iASPP potentiates its ability to inhibit p53 and NF-κB
Source: Oncotarget. 2015 Dec 5;6(40):42478–90. doi: 10.18632/oncotarget.6478 (PMC4767446; doi:10.18632/oncotarget.6478)
Supplement: Supplementary file 1 [file oncotarget-06-42478-s001.pdf]

Caspase cleavage of iASPP potentiates its ability to inhibit p53 and NF-κB

Supplementary Material

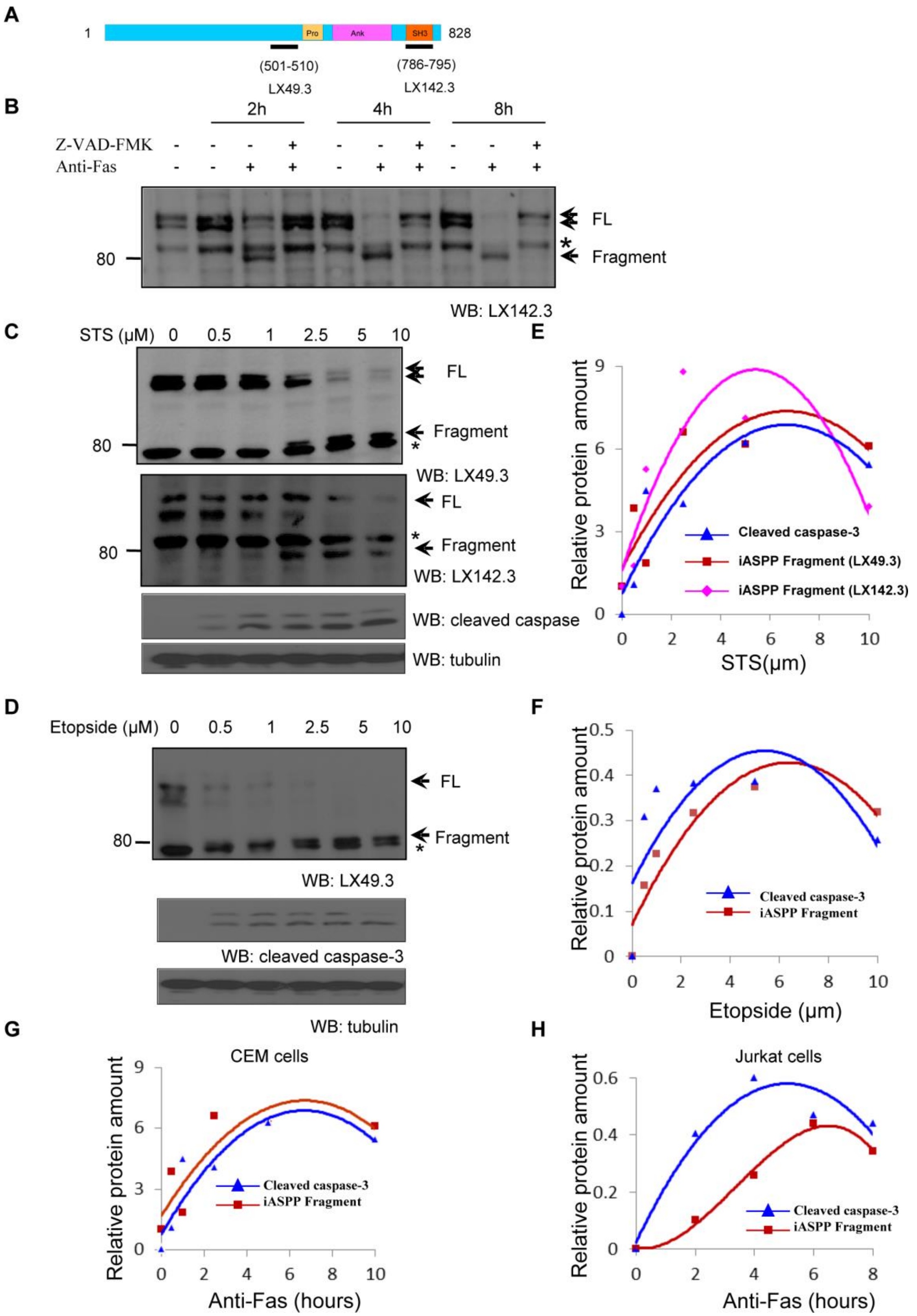

Supplementary Figure S1: Caspase-induced iASPP cleavage in response to DNA damage reagents. A. Schematic representation of the iASPP protein and locations of residues recognized by anti-iASPP antibodies. **B.**

Jurkat cells were exposed to anti- Fas antibody (250ng/mL) for the indicated period of time. To inhibit caspase activity, cells were pre-incubated with 25μM z-VAD-FMK for 1 hour. iASPP cleavage was detected by western blotting with LX142.3 antibody. The asterisk indicates a band recognized by one anti-iASPP antibody but not by another, which may result from non-specific cross-reaction with other proteins. Jurkat cells were stimulated to undergo apoptosis by treatment with Staurosporine (STS) **C.** or etopside **D.** for 4 hours. iASPP cleavage was examined by western blotting with anti-iASPP antibody LX49.3. The arrowheads indicate iASPP or the iASPP cleavage product of about 80kDa. Tubulin was used as a loading control. Cleaved caspase-3 was used as a marker to confirm the activation of caspases after the treatment. Protein quantification was performed with Imagine J software. The dot graphs (E-H) show the trend of iASPP cleavage and caspase activation upon STS **E.**, etopside **F.**, or anti-Fas treatment in CEM **G.** and Jurkat **H.** cells.

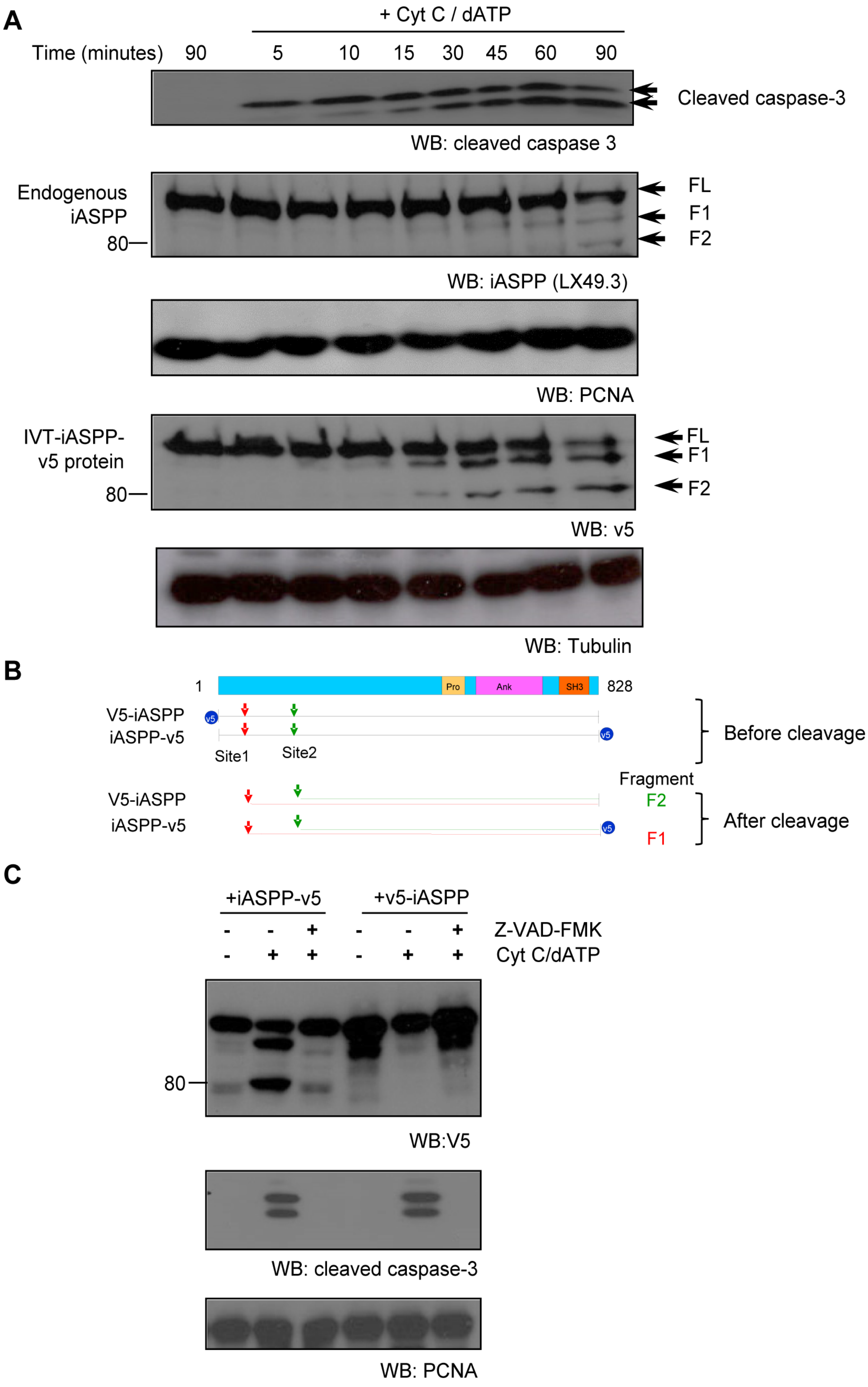
A
+ Cyt C / dATP
Time (minutes) 90 5 10 15 30 45 60 90
Cleaved caspase-3
WB: cleaved caspase 3
Endogenous iASPP
80
FL
F1
F2
WB: iASPP (LX49.3)
WB: PCNA
IVT-iASPP-v5 protein
80
FL
F1
F2
WB: v5
WB: Tubulin
B
1
Pro
Ank
SH3
828
V5-iASPP
iASPP-v5
v5
v5
Site1
Site2
Before cleavage
Fragment
V5-iASPP
iASPP-v5
F2
F1
After cleavage
C
+iASPP-v5
+v5-iASPP
- - + - - + Z-VAD-FMK
- + + - + + Cyt C/dATP
80
WB:V5
WB: cleaved caspase-3
WB: PCNA

Supplementary Figure S2: Time-dependent iASPP cleavage *in vivo* at its N-19 **terminus A.** Cell-free extracts from CEM cells were generated. Cyt c and dATP were added and the reactions performed at 37°C, for the indicated time periods, to trigger caspase activation. Cleavage of endogenous iASPP or *in vitro* translated C- terminally v5 tagged iASPP that was added to apoptotic cell-free extracts were detected with anti-iASPP antibody LX49.3 and anti-v5 tag antibody, respectively. PCNA or β-tubulin were used as loading controls to confirm that the same amount of cell-free extracts had been added into the reaction system. The arrowheads indicate iASPP and iASPP cleavage products of about 80kDa and 95kDa. **B.** Schematic representation of iASPP, truncated iASPP and the predicted iASPP caspase cleavage products. At the top of the diagram, FL-iASPP is schematically represented with its functional domains. Below this, the full length iASPP-v5, v5-iASPP and iASPP truncated products are shown as lines. The arrowheads indicate the potential iASPP cleavage sites responsible for the production of 95kDa (red) and 80kDa (green) iASPP fragments from full length iASPP. SH, Src homology domains; Pro, proline rich domain; Ank, ankyrin repeats. **C.** iASPP is cleaved at C-terminus. FL-iASPP- v5, tagged at the C-terminus (iASPP-v5) was translated *in vitro*. The same amounts of products were mixed with apoptotic CEM cell-free extract, triggered by adding cytochrome c and dATP. The reactions were performed at 37°C for 1 hour. z-VAD- FMK was added as indicated to inhibit apoptosis. The proteins were separated by 8% SDS-PAGE and blotted with anti-v5 antibody.

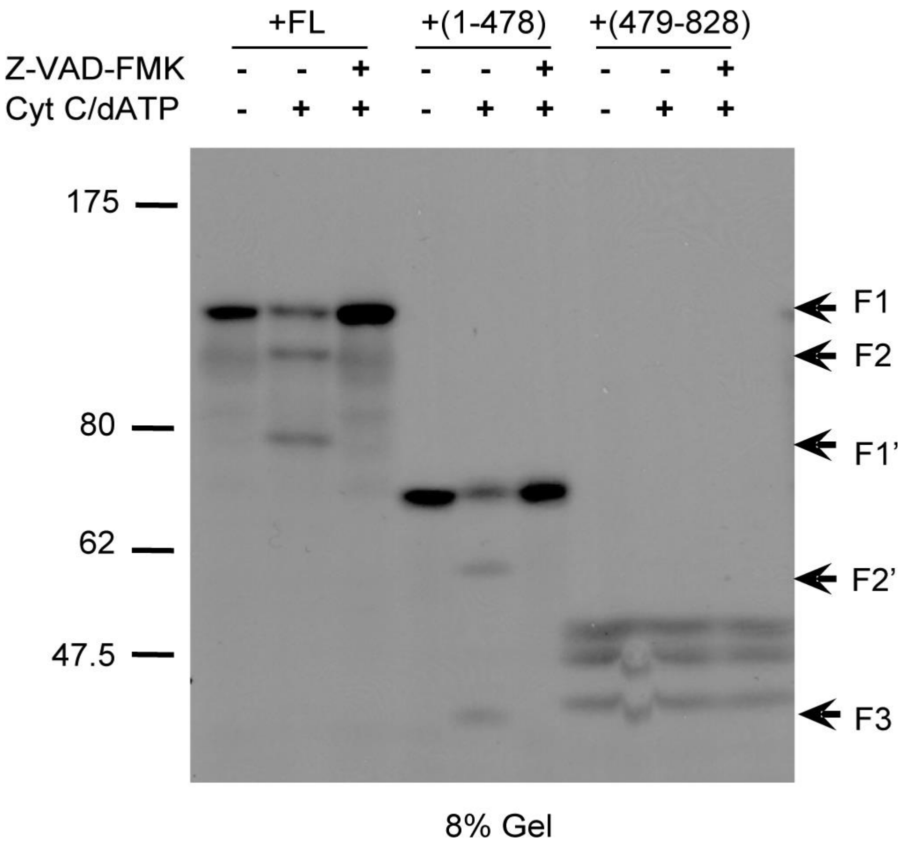

Supplementary Figure S3: Putative iASPP cleavage sites at D91 and D294. I*n vitro* translated FL-iASPP, iASPP(1-478), and (479-828) were labelled with [35S] methionine and mixed with cell-free extracts in the presence or absence of Cytochrome c (Cyt C) and dATP. The presence of radiolabelled iASPP and iASPP fragments, separated on an 8% PAGE gel, were detected by autoradiography.
